# Metaplastic Breast Carcinoma: A Rare Presentation in a Young Female With Double Hormone Receptor-Positive Tumor

**DOI:** 10.7759/cureus.65674

**Published:** 2024-07-29

**Authors:** Anas Al Rabadi, Ahmad Al Kofahi, Laith Albudour, Mansour Abushqair, Taher Harahsheh, Hend Al-harahsheh, Duaa Al-shurbaji

**Affiliations:** 1 Department of Surgery, Jordanian Royal Medical Services, Amman, JOR; 2 Department of Surgery, Royal Medical Services, Amman, JOR; 3 Department of Plastic and Reconstructive Surgery, King Hussein Medical City, Amman, JOR; 4 Department of General Surgery, Royal Medical Services, Amman, JOR; 5 Department of Radiology, Royal Medical Services, Amman, JOR

**Keywords:** mammogram screening, invasive ductal breast carcinoma, immunohistochemistry staining, breast cancer research, metaplastic breast carcinoma (mbc)

## Abstract

Metaplastic breast carcinoma (MBC) represents a rare subtype of breast cancer, characterized by poor prognostic indicators that have been recently identified. Clinical and radiological presentations often mimic other breast cancer types, necessitating immunohistochemistry (IHC) for accurate diagnosis. In this study, we report a case involving a 31-year-old female presenting with a painless, fixed, non-inflammatory mass in the left breast, which was confirmed as MBC. Treatment encompassed lumpectomy, chemotherapy, radiotherapy, and subsequent hormonal therapy. Understanding this rarely reported yet aggressive form of breast cancer holds significant importance for clinicians, enabling them to promptly establish a diagnosis and implement effective management strategies.

## Introduction

Metaplastic breast carcinoma (MBC) is a rare subtype of invasive breast cancer consisting histologically of a mixed composite of mesenchymal and epithelial malignant features. It accounts for 0.2%-1% of all breast carcinomas, which, due to its rarity, has not been identified as a separate entity until 2000 [1]. Although it is exceedingly rare, the poor prognostic factors associated with this cancer have led to an increasing interest in recognizing a better way of managing such cases, especially after showing resistance to usual hormonal therapy due to its triple negativity [2].

MBCs are recognized as a subset of ductal carcinoma, typically displaying diverse heterogeneous characteristics [2], with a median age of presentation ranging from 48 to 59 [3]. The poor prognosis of this tumor is linked to its distinctive features, such as a mass typically exceeding 2 cm in size and showing rapid growth [3]. Histologically, these lesions display microscopic calcification and can manifest as a blood-rich lesion [4].

Diagnosing MBC presents challenges due to its wide array of imaging and clinical presentations. Radiologists and clinicians often lack extensive experience in diagnosing and managing MBC, as it is a rare condition with limited documentation in the literature [3].

We herein report a rare case of MBC in a 31-year-old female patient to underscore the distinct features of this tumor, which can be mistaken for other subgroups of breast cancer. By elucidating these characteristics, we aim to enhance comprehension and facilitate tailored management strategies, ultimately improving long-term outcomes for MBC patients.

## Case presentation

A 31-year-old Jordanian female presented to the department of surgery clinic complaining of a painless, non-mobile mass in the left breast of three months duration. Upon initial physical examination, the mass was found to be painless, fixed, and palpable without any signs of inflammation, discharge, swelling, nipple retraction, changes in skin color, or recent unintentional weight loss. The mass appeared fixed to the underlying tissue, without tenderness, redness, or dilated veins.

**Figure 1:**
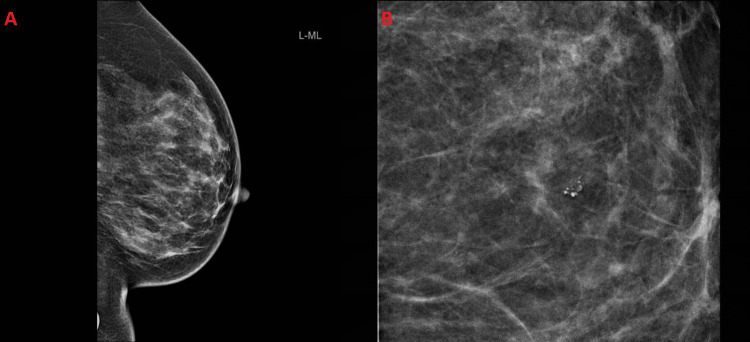
Mammogram showing a poorly defined isodense mass located in the lower outer quadrant of the left breast (A). Multiple calcifications grouped in the mid-inner aspect of the left breast (B).

Radiological examinations included a computed tomography (CT) scan, which indicated the absence of visceral metastasis and no evidence of bone metastasis on the bone isotope scan. Additionally, a bilateral mammogram was conducted with mediolateral oblique (MLO) and craniocaudal (CC) views. The findings were indicative of a highly suspicious malignancy, as illustrated in Figure 1A-1B. The mammogram revealed a poorly defined isodense mass located in the left breast's lower outer quadrant, along with multiple calcifications grouped in the mid-inner aspect of the left breast, approximately 3.5 cm away from the nipple. Asymmetric densities were observed in both breasts, with relative compression evident on spot compression views.

On breast ultrasonography, the left breast's lower outer quadrant mass appeared lobulated, hypoechoic, and partially irregular, measuring around 2.7 × 1.7 cm. Left axillary lymph node cortices appeared thickened, and some appeared enlarged. In conclusion, the mass was classified according to Breast Imaging Reporting and Data System (BI-RADS) as 4c. Fine needle aspiration was done for the lymph nodes, but the results were not accurate as it was an insufficient sample.

**Figure 2:**
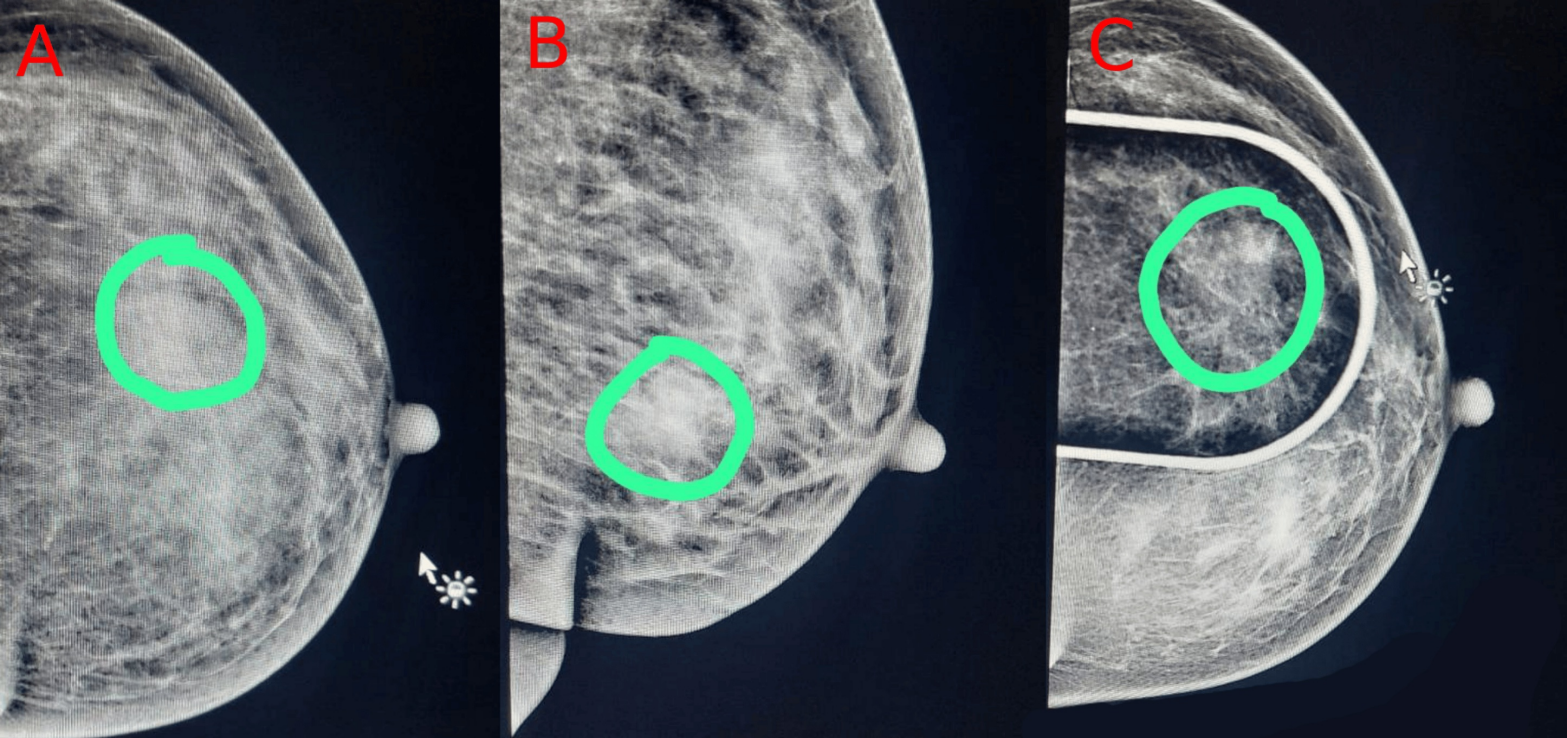
MLO aspect (circles refer to the mass). MLO: mediolateral oblique

The patient underwent left breast-wide local excision with left axillary nodal dissection for sampling, and a specimen was sent to the histopathological department. On gross examination, the specimen measured 8 × 6 × 3 cm covered partially by a skin ellipse (4 × 2 cm). The cut surface shows a lobulated, whitish, hard mass measuring 2.3 × 2 × 1.5 cm located 1 mm away from the anterior surgical excision margin. There were multiple fatty fragments measuring in aggregate 7 × 4 cm containing nine lymph nodes. The MLO view is shown in Figure 2.

**Figure 3:**
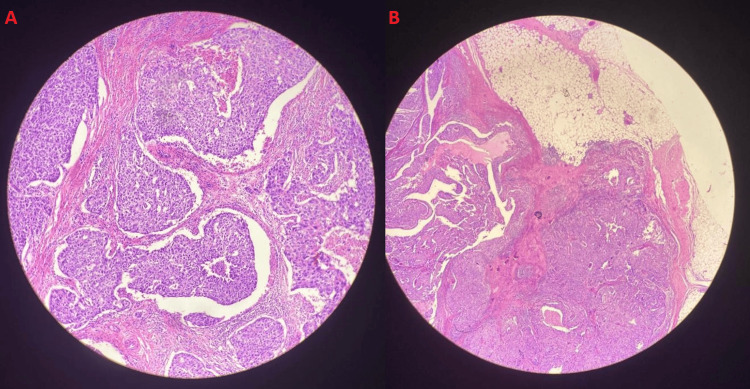
Microscopic examination revealed findings consistent with metaplastic breast carcinoma (hematoxylin and eosin, magnification ×20).

The histopathological report of the biopsy showed metaplastic carcinoma, located 1 mm away from the anterior surgical excision margin, with a histological grade of 3, indicating poorly differentiated cells. There was no tubular differentiation (score 3), and there was marked nuclear pleomorphism (score 3), with a mitotic count of more than 20 per 10 high power field also seen (score 3), resulting in an overall 9/9 on Nottingham score, resembling high-grade cancer. According to the American Joint Committee on Cancer's eighth edition classification system, the tumor was classified as pT2N1aM0. Ductal carcinoma in situ (DCIS) was also present, with a high nuclear grade, consisting of solid and comedo subtypes. The overlying skin was present and unaffected. Upon examination of the nine lymph nodes, involvement was detected in one, characterized by macrometastasis. The illustrations are shown in Figure 3A-3B.

Immunohistochemistry (IHC) staining conducted on the tru-cut biopsy revealed a double hormone receptor-positive breast cancer, with the following results: estrogen receptors were positive, progesterone receptors were strongly positive, and HER2 was negative. Additionally, CK5/6 showed diffuse positivity, while Ki67 was positive. CK14, synaptophysin, and chromogranin were all negative.

In the adjacent breast tissue to the mass, fibrocystic change and flat epithelial atypia were observed. A dense lymphoid cell infiltrate was associated with the tumor, as well as the benign terminal duct lobular units.

For management, the patient underwent adjuvant chemotherapy consisting of four cycles of Adriamycin and cyclophosphamide (AC), followed by four cycles of docetaxel. After completing six months of chemotherapy, adjuvant external beam radiation therapy (EBRT) was initiated. Additionally, hormonal therapy with tamoxifen and goserelin for a six-month duration was started.

**Figure 4:**
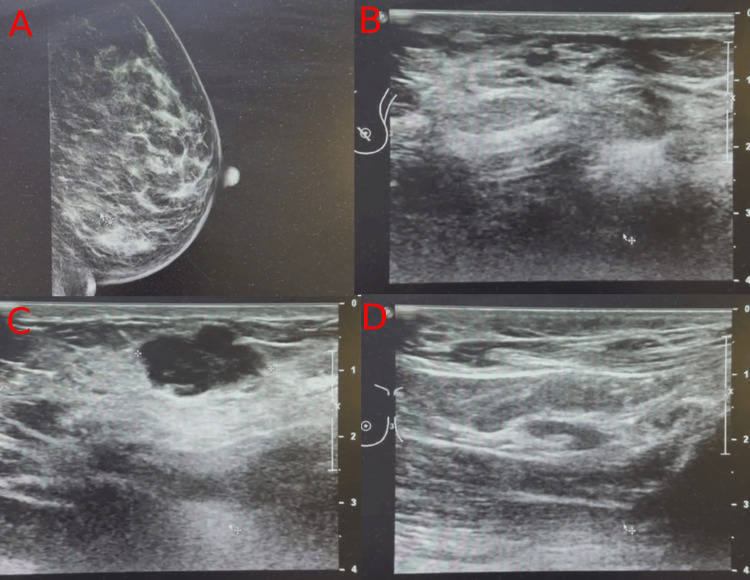
Follow-up mammogram (A) and ultrasound imaging (B-D).

During follow-up, an ultrasound examination revealed the absence of axillary lymph node metastasis. Subsequent follow-up appointments were scheduled every 4-6 months for breast and axilla ultrasound, mammogram, and laboratory checkups (Figure 4).

## Discussion

MBC manifests as a rapidly growing palpable mass, representing a subtype of invasive ductal carcinomas (IDCs). It is characterized by being a poorly differentiated heterogeneous tumor containing ductal carcinoma cells mixed with areas of spindle, squamous, chondroid, or osseous elements. However, its prognosis is notably poorer compared to IDC, as it exhibits a poor response to neoadjuvant therapy. Furthermore, postoperative chemotherapy has not demonstrated significant success, making surgery the primary treatment option [5,6].

Several theories exist regarding the pathogenesis of MBC. Some researchers propose that it may arise from epithelial to mesenchymal transition, while others suggest that its origin lies with cancer stem cells or myoepithelial progenitor cells [7]. The mesenchymal differentiation observed in metaplastic tumors exhibits various patterns and combinations, with atypia ranging from bland to extremely malignant features, which serve as indicators of poor prognosis [8]. Conversely, when the predominant features consist of carcinoma-like components such as benign heterologous components and absent intervening stroma, these suggest a better prognosis [9].

Histologically, MBC exhibits a broad spectrum of microscopic appearances, leading to various classifications. The World Health Organization (WHO) categorizes it into epithelial type and mixed type, each with further subtypes. Additionally, the Wargotz classification identifies MBC as matrix-producing carcinoma, squamous cell carcinoma, spindle cell carcinoma, carcinosarcoma, and metaplastic carcinoma with osteoclastic giant cells [3]. These variations in histological morphology pose challenges in both diagnosis and treatment.

Metaplastic breast carcinoma presents a diverse array of histological patterns, which can result in a wide-ranging list of potential diagnoses. Typically, a combination of morphology assessment and immunohistochemical staining for high-molecular-weight cytokeratins and p63 is employed to establish a diagnosis [8]. Immunohistochemical confirmation of the tumor epithelial component is essential for diagnosing metaplastic carcinoma. Commonly used markers include CK5/6, CK14, CK(AE1/AE3), and 34βE12. Immunopositivity for p63 and CK is frequently observed in most MBCs, distinguishing them from fibromatosis [4].

On a molecular level, MBC typically presents as a triple-negative tumor (TN), as it often lacks expression of estrogen receptor (ER), progesterone receptor (PR), and human epidermal growth factor 2 (HER2), accounting for approximately 90% of cases [10]. However, a small percentage (8%) of MBCs are classified as double-positive tumors (ER+, PR+), rendering them considerably rare [11].

The molecular characteristics of this tumor make it challenging to treat. In comparison to other breast cancers, there are fewer therapeutic options available, and outcomes tend to be poorer, often associated with a higher recurrence rate [12]. Previous studies suggest that MBC exhibits a poor response to systemic chemotherapy, and neoadjuvant chemotherapy has limited efficacy in halting its progression [12]. Aggressive surgical interventions, often involving wide local excision of the lesion, are frequently necessary due to the high recurrence rate associated with MBC. However, there is no consensus on the specific surgical margins required. Surgery remains the primary treatment modality for MBC. Adjuvant radiotherapy can contribute to reducing the rate of recurrence and mortality [9]. Nonetheless, certain studies have identified MBCs as chemoresistant, posing additional challenges in treatment [9].

Despite the rarity of MBC and the limited available data, particularly in younger populations, our findings emphasize the importance of considering MBC even in such cases. While MBC typically presents as a triple-negative tumor, our case highlights the existence of alternative forms, including double-positive tumors, warranting further investigation into their clinical distinctions. Although various treatment modalities have been explored, surgery remains the cornerstone of MBC management. Further research is essential to delineate optimal therapeutic strategies and improve outcomes for MBC patients.

In conclusion, our study underscores a unique case of MBC in a young 31-year-old patient, distinguished by its double-positive histological profile. By elucidating the distinctive characteristics of this tumor, often confused with other breast cancer subgroups, we aim to deepen comprehension and guide tailored treatment approaches.

## Conclusions

In conclusion, our study underscores a unique case of MBC in a young 31-year-old patient, distinguished by its double-positive histological profile. By elucidating the distinctive characteristics of this tumor, often confused with other breast cancer subgroups, we aim to deepen comprehension and guide tailored treatment approaches.
